# Genome Sequence and Assembly of 18 Fusarium Isolates from Florida Citrus under High Huanglongbing Disease Pressure and California Citrus under Low Huanglongbing Disease Pressure

**DOI:** 10.1128/mra.00101-23

**Published:** 2023-04-12

**Authors:** Tania Kurbessoian, Gretchen Heimlich-Villalta, Nichole Ginnan, Flavia Campos Vieira, Philippe E. Rolshausen, M. Caroline Roper, Jason E. Stajich

**Affiliations:** a Department of Microbiology and Plant Pathology, University of California, Riverside, California, USA; b Department of Botany and Plant Sciences, University of California, Riverside, California, USA; c Institute for Integrative Genome Biology, University of California, Riverside, California, USA; Vanderbilt University

## Abstract

The genomes of eighteen Fusarium isolates cultured from diseased and healthy citrus trees were sequenced, assembled, and annotated. Isolate species identification was confirmed using single marker (TEF1-alpha) phylogenetic assessment. Studies of the traits and genotypes of plant-associated isolates are important to understanding the fungal contribution to phytobiomes of citrus.

## ANNOUNCEMENT

Fungal isolates were cultured from leaf, stem, and root tissue of California (Riverside County) and Florida (Marion, Lake, and Martintown Counties) citrus trees. Samples were taken from *Citrus* sp. scions grafted onto Citrus aurantium and × *Citroncirus* species rootstocks. California trees had low Huanglongbing (HLB) disease pressure; Florida trees had high HLB pressure. Fungal taxonomy was initially assigned by internal transcribed spacer (ITS) sequencing (1) and confirmed using single marker phylogenetic analysis.

Strains were grown on potato dextrose agar (PDA) for 1 week, collected by scraping fungal mycelial tissue from the medium, and frozen in liquid nitrogen. High-molecular-weight DNA was extracted from fungal tissue based on reference 2. Genomic libraries for the 18 isolates were constructed with the Illumina DNA Prep kit with 10-bp IDT UDI indices and sequenced on an Illumina NextSeq 2000 sequencer in 2- by 151-bp paired-end format at the MiGS sequencing center (Pittsburgh, PA). Reads were trimmed and demultiplexed by the bcl-convert workflow to produce Fastq files. Two strains (Fusarium oxysporum CF00159 and Fusarium falciforme CF00175) were additionally sequenced with Oxford Nanopore Technologies (ONT) (3) at MiGS. An average of 5.1 million Illumina reads and 0.5 million ONT reads were produced (Table 1).

**TABLE 1:** Strain and species designation, isolation source, sequencing read, assembly, and annotation statistics, and accession numbers^*d*^

Species	Strain ID^*e*^	Host/tissue	Location	GenBank accession no.	SRA accession no.	No. of read pairs	Coverage	No. of contigs	Genome size (Mbp)	Contig *L*_50_	Contig *N*_50_ (kbp)	G+C content (%)	Genome completion (BUSCO %)	No. of genes	Telomeres found^*f*^
F. solani	CF00177	*Citrus jambhiri* Lush. (Schaub rough lemon),	Riverside County, CA	JAOQAT000000000	SRR21444562	5,208,997	28.56	1,595	54.7	29	560	50.61	99.8	17,742	8F, 7R
*F. falciforme*	CF00178	*Citrus jambhiri* Lush. (Schaub rough lemon),	Riverside County, CA	JAOQAU000000000	SRR21444563	4,824,837	24.65	1,010	56.4	32	504	49.12	99.8	15,807	3F, 4R
*F. falciforme*	CF00179	*Citrus jambhiri* Lush. (Schaub rough lemon),	Riverside County, CA	JAOQAV000000000	SRR21444564	6,299,623	33.75	866	54.3	23	663	49.35	99.9	14,964	1F, 1R
*F. falciforme*	CF00180	*Citrus aurantifolia* Christm. (Mexican lime)/x *Cintroncirus* spp. (Swingle),	Riverside County, CA	JAOQAW000000000	SRR21444565	6,641,869	34.75	1,035	55.3	27	595	49.49	99.9	14,885	4F, 3R
*F. falciforme*	CF00175	Citrus sinensis L. Osbeck (Parson Brown)/*Citrus aurantium* (sour orange), root	Marion County, FL	JAOQBJ000000000	SRR21444571	5,036,413	39.66 (24.4)^*a*^	296	65.5	9	2,609	47.69	98.2	16,794	1F, 3R
*F. falciforme* (ONT)	CF00175	*Citrus sinensis* L. Osbeck (Parson Brown)/*Citrus aurantium* (sour orange), root	Marion County, FL		SRR21444561	674,410^*b*^	15.26				6.831^*c*^				
*F. equiseti*	CF00095	*Citrus sinensis* L. Osbeck (Parson Brown)/*Citrus aurantium* (sour orange), stem	Lake County, FL	JAOQBH000000000	SRR21444559	4,858,320	37.09	89	37.1	8	1,904	47.76	99.9	12,206	4F, 8R
*F. irregulare*	CF00137	*Citrus sinensis* L. Osbeck (Parson Brown)/*Citrus aurantium* (sour orange), stem	Marion, County, FL	JAOQBA000000000	SRR21444569	6,189,289	46.7	41	37.9	8	1,635	48.05	99.8	12,502	9F, 6R
*F. irregulare*	CF00143	*Citrus sinensis* L. Osbeck (Parson Brown)/*Citrus aurantium* (sour orange), stem	Marion County, FL	JAPDHF000000000	SRR21444553	4,877,712	36.52	42	38.1	9	1,635	48.03	99.8	12,865	10F, 5R
F. oxysporum	CF00115	*Citrus sinensis* L. Osbeck (Hamlin)/× *Citroncirus* spp. (Swingle), leaf	Lake County, FL	JAOQAX000000000	SRR21444566	4,443,693	27.44	208	45.0	13	1,348	47.41	94.7	15,474	0F, 2R
F. oxysporum	CF00132	*Citrus sinensis* L. Osbeck (Parson Brown)/*Citrus aurantium* (sour orange), root	Marion County, FL	JAOQAY000000000	SRR21444567	5,666,638	33.92	383	48.6	11	1,590	47.63	99.9	16,669	3F, 3R
F. oxysporum	CF00141	*Citrus sinensis* L. Osbeck (Parson Brown)/*Citrus aurantium* (sour orange), leaf	Marion County, FL	JAOQBB000000000	SRR21444552	4,806,668	29.53	239	45.0	14	988	47.44	94.6	15,308	12F, 8R
F. oxysporum	CF00144	*Citrus sinensis* L. Osbeck (Parson Brown)/*Citrus aurantium* (sour orange), leaf	Marion County, FL	JAOQBC000000000	SRR21444554	3,961,972	25.02	107	42.2	8	1,593	47.52	92.2	14,579	13F, 12R
F. oxysporum	CF00145	*Citrus sinensis* L. Osbeck (Parson Brown)/*Citrus aurantium* (sour orange), leaf	Marion County, FL	JAOQBD000000000	SRR21444555	4,867,827	30.81	60	42.9	7	2,090	47.5	92.3	14,883	13F, 9R
F. oxysporum	CF00159	*Citrus sinensis* L. Osbeck (Parson Brown)/*Citrus aurantium* (sour orange), root	Marion County, FL	JAOQBI000000000	SRR21444570	4,130,577	43.7 (24.7)^*a*^	136	50.6	11	1,462	47.38	98.9	17,802	13F, 7R
F. oxysporum (ONT)	CF00159	*Citrus sinensis* L. Osbeck (Parson Brown)/*Citrus aurantium* (sour orange), root	Marion County, FL		SRR21444560	391,895^*b*^	19.03				5.912^*c*^				
F. oxysporum	CF00160	*Citrus sinensis* L. Osbeck (Parson Brown)/*Citrus aurantium* (sour orange), leaf	Marion County, FL	JAOQBE000000000	SRR21444556	4,257,800	26.27	259	44.9	11	1,126	47.41	94.1	15,276	12F, 8R
F. oxysporum	CF00161	*Citrus sinensis* L. Osbeck (Parson Brown)/*Citrus aurantium* (sour orange), leaf	Marion County, FL	JAOQBF000000000	SRR21444557	6,329,714	40.21	59	41.9	9	1,492	47.56	92.3	14,538	10F, 9R
F. oxysporum	CF00165	*Citrus sinensis* L. Osbeck (Hamlin)/× *Citroncirus* spp. (Swingle), leaf	Lake County, FL	JAOQBG000000000	SRR21444558	3,918,330	23.96	245	44.9	11	1,552	47.38	93.6	15,470	0F, 3R
*F. torreyae*	CF00136	*Citrus sinensis* L. Osbeck (Valencia)/× *Citroncirus* spp. (Swingle), leaf	Martintown County, FL	JAOQAZ000000000	SRR21444568	6,378,752	39.89	92	46.5	13	1,014	47.92	99.8	14,845	17F, 17R

a Summary statistics shown are for hybrid genome assembly with Illumina and ONT sequence reads.

b ONT reads are single ended.

c *N*_50_ for ONT read lengths.

d Host/tissue indicates host material from which strain was isolated. Location indicates United States location, either from California (CA) or from Florida (FL); full location description is available in NCBI BioSample. The GenBank accession number of the deposited genome assembly and the SRA accession number for individual sequencing runs are listed for each isolate. The number of reads was used to help determine the coverage values for almost all the genomes except the two Nanopore genomes mentioned. Oxford Nanopore Technologies (ONT) sequencing data coverage is calculated using average depth of sequencing for only Nanopore reads. Genomes CF159 and CF175 had combined Illumina and ONT coverage calculated and indicated in the table, where Illumina-only coverage is in parentheses next to the total coverage. Genome assembly calculations include number of contigs, genome size, *N*_50_ (longest in length in 50% of genome), *L*_50_ (number of contigs that are longest in length in 50% of genome), and G+C content, while genome annotation results include number of genes predicted and annotated. BUSCO completion statistics and comparisons were determined using the sordariomycetes_odb10 database with 3,817 genes. Telomeres were calculated on completed genomes using find_telomere.py script (41).

e ID, identifier.

f F, forward; R, reverse.

Genome assembly of Illumina reads was performed using AAFTF (4–8) for performing filtering and trimming steps for data quality and SPAdes (3.15.4) (9) for assembly. Default parameters for the underlying tools were applied throughout. Assembly of the two ONT-sequenced isolates was performed using Canu (v.2.2) (10) and Flye (v.2.9-b1774) (11), followed by assembly polishing with Medaka (v.1.6) (12). Both Nanopore assemblies were processed with five rounds of polishing by Pilon (v1.24) (13) Canu (CF159) and Flye (CF175) assemblies with Illumina sequencing reads via the AAFTF ‘pilon’ step. Contigs were reordered and renamed from largest to smallest with the ‘sort’ command. Assembly summary statistics were calculated with the ‘assess’ tool in AAFTF and genome completeness by BUSCO (v5.2.2) (14) with the sordariomycetes_odb10 database of 3,817 marker genes. Genome annotation was performed with Funannotate (v.1.8.10) (15–32) using default parameters for the underlying tools applied throughout. Genome sequencing, assembly, and protein coding gene annotation statistics of the 18 genomes are summarized in Table 1.

**FIG 1:**
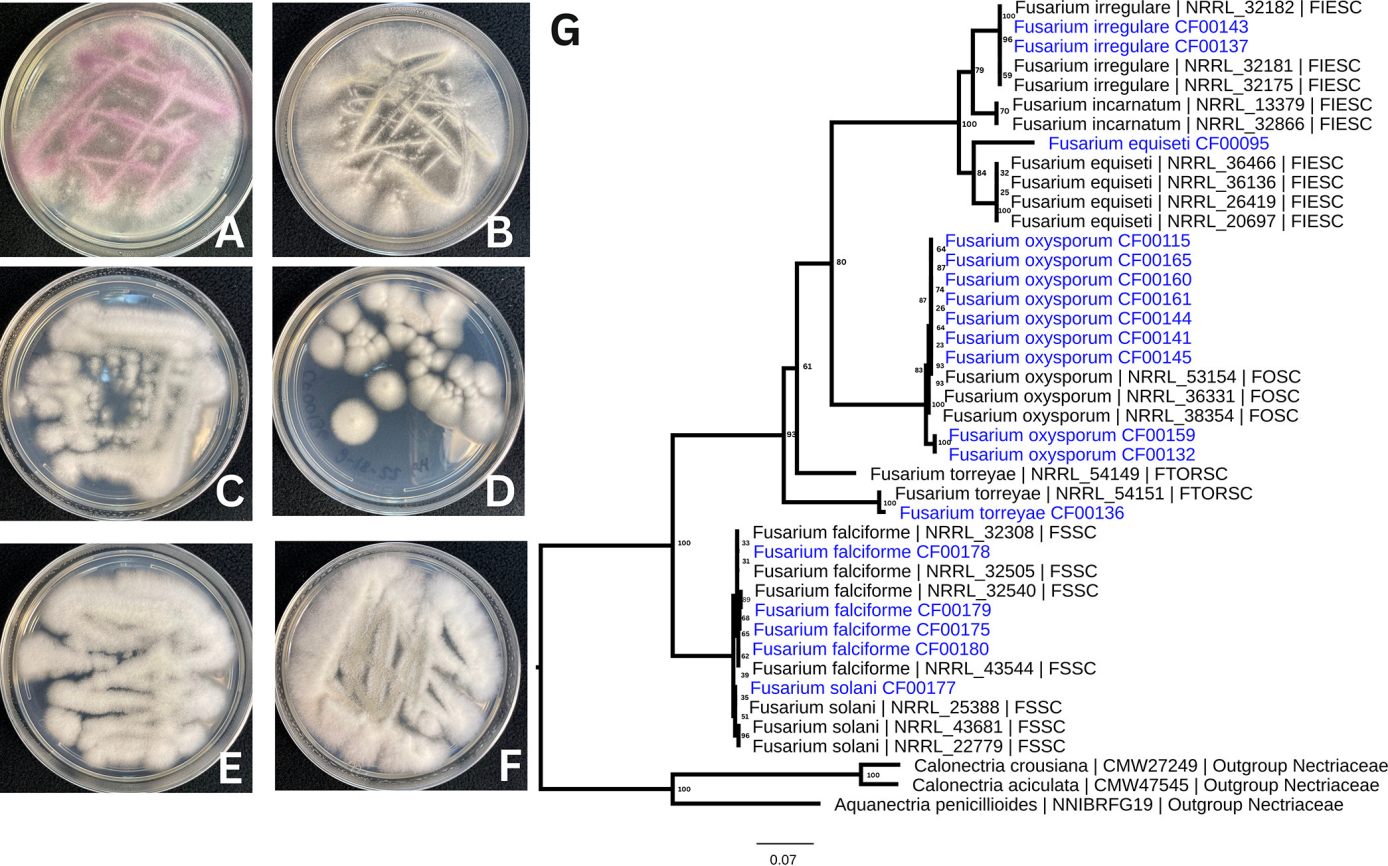
Agar culture of Fusarium species isolated from citrus and phylogenetic tree describing 18 strains with respective NCBI isolates. (A) Isolate CF115, Fusarium oxysporum. (B) Isolate CF00179, Fusarium falciforme. (C) Isolate CF00136, Fusarium torreyae. (D) Isolate CF00137, Fusarium irregulare. (E) Isolate CF00177, Fusarium solani. (F) Isolate CF00095, Fusarium equiseti. (G) Species assignments were inferred from the phylogenetic tree constructed from TEF1 sequences for the 18 isolates from this study (blue) and 21 reference sequences of identified Fusarium species and rooted with sequences of three outgroup taxa. Identification abbreviations for Fusarium species complexes are FIESC (Fusarium incarnatum*-equiseti* complex), FOSC (Fusarium oxysporum species complex), FTORSC (Fusarium torreyae species complex), and FSSC (Fusarium solani species complex).

BLASTN was used to capture translation elongation factor 1 (TEF1) (MG183712) sequences of each genome assembly for species identification (33–35). A multiple sequence alignment of identified TEF1 genes and those available in FUSARIUM-ID v 3.0 (36) was created with MUSCLE (5.1) (37). The alignment was trimmed with ClipKIT (38), and the phylogenetic relationships of the strains were inferred with IQ-TREE 2 (39). The 18 isolates were placed among six known Fusarium species (Fig. 1), and their position was used to assign the taxonomic identification presented in Table 1.

### Data availability.

This whole-genome project has been deposited at DDBJ/ENA/GenBank under the BioProject accession no. PRJNA855134. The individual SRA read accession numbers and genome accession numbers for each isolate are listed in Table 1. Genome assembly, annotation, and TEF1 phylogenetic assessment pipeline and related code are archived in Zenodo (40).
